# Prevalence, severity, and correlates of post-traumatic stress disorder in 205 outpatients with schizophrenia: results from a cross-sectional, observational study assessing diagnoses via the Mini-International Neuropsychiatric Interview

**DOI:** 10.1017/S1092852926101035

**Published:** 2026-06-17

**Authors:** Julieta Ramirez, María Delfina Tavella, German Weisbrot, Ricardo Corral, Adrian Cabrera, Alejo Corrales, José Gramajo, Nora Crespi, Christoph U. Correll

**Affiliations:** 1 Department of Psychiatry, José T. Borda Hospital, Argentina; 2 Argentine Association of Psychiatry (AAP), Buenos Aires, Argentina; 3 Argentine Association of Biological Psychiatry (AAPB), Buenos Aires, Argentina; 4 José T. Borda Hospital, Argentina; 5 University of Buenos Aires, Argentina; 6 National University of Tucuman, Argentina; 7 Department of Psychiatry, Zucker Hillside Hospital, Northwell Health, Glen Oaks, NY, USA; 8 Department of Psychiatry and Molecular Medicine, Donald and Barbara Zucker School of Medicine at Hofstra/Northwell, Hempstead, NY, USA; 9 Department of Child and Adolescent Psychiatry, Charité Universitätsmedizin Berlin, Berlin, Germany; 10 German Center for Mental Health (DZPG), Partner Site Berlin, Berlin, Germany; 11 Einstein Center for Population Diversity (ECPD), Berlin, Germany; 12 Einstein Center for Youth Mental Health (ECYM), Berlin, Germany

**Keywords:** Schizophrenia, post-traumatic stress disorder, frequency, correlates, inflammatory markers

## Abstract

**Objectives:**

To report prevalence, severity and clinical and inflammatory correlates of Post-Traumatic Stress Disorder (PTSD) in patients with schizophrenia.

**Methods:**

This cross-sectional, observational study included adult outpatients with schizophrenia/schizophreniform disorder. Assessments included the Mini-International Neuropsychiatric Interview (MINI), Post-traumatic Stress Disorder Symptom Severity Scale-Revised, Positive and Negative Syndrome Scale (PANSS), Calgary Depression Scale for Schizophrenia (CDSS), Global Assessment of Functioning Scale (GAF), and inflammatory markers (neutrophil-to-lymphocyte ratio, and platelet-to-lymphocyte ratio, systemic immune-inflammation index: platelets × neutrophils)/lymphocytes). Analyses compared patients with (SCZ + PTSD) versus without PTSD (SCZ-PTSD) across demographic, clinical, and biological variables.

**Results:**

SCZ + PTSD had higher PANSS-total scores (80.7 ± 19.5 vs. 71.6 ± 17.1; p = 0.003), more comorbid depression (73.8% vs. 23.3%; p < .001), higher CDSS scores (9.5 ± 4.9 vs. 3.2 ± 4.1; p < .001), more frequent suicide attempts (40.5% vs. 23.0%; p = 0.05), lower GAF scores (39.8 ± 12.3 vs. 49.6 ± 12.3; p < .001) and more medical comorbidities (51.1% vs. 42.8%; p = .014). Inflammatory markers did not differ significantly between groups. Logistic regression analyses identified higher CDSS scores (OR = 1.25, 95% CI [1.15, 1.36], p < .001) and lower GAF scores (OR = 0.96, 95% CI [0.93, 0.99], p = .007) as significant correlates of PTSD (r^2^ = 0.22).

**Conclusions:**

PTSD is common and clinically relevant in people with schizophrenia, underscoring the need for routine screening and targeted interventions in this population.

## Introduction

Schizophrenia is a severe mental disorder that affects approximately 1% of the population worldwide.^1^ Compared to the general population, patients with schizophrenia have an increased prevalence of psychiatric comorbidities, including anxiety disorders, depression, substance-abuse disorder, obsessive-compulsive disorder, and post-traumatic stress disorder (PTSD).^2^ ^,^^3^ ^,^^4^

PTSD is a condition of persistent mental and emotional stress as a result of exposure to a traumatic event. PTSD symptoms include severe anxiety and intrusive thoughts about the traumatic event.^5^ ^,^^6^ PTSD frequently co-occurs with schizophrenia.^7^ ^,^^8^ The first study to assess the prevalence of PTSD in people with schizophrenia was published in the late 1980s.^9^ However, the prevalence of PTSD in schizophrenia is still somewhat uncertain, ranging from 10 to 30% in most studies.^7^ ^,^^10^ ^,^^11^ ^,^^12^ ^,^^13^

Nevertheless, these frequencies of PTSD, which are influenced by differences in sample recruitment and composition, are much higher than in the general population, which ranges from 7.8 to 9.2%.^6^ ^,^^14^

Approximately 70–98% of patients with schizophrenia have experienced a traumatic event in their life,^12^ ^,^^15^ compared to 39–56% in the general population.^6^ ^,^^16^ Complex trauma histories are common among people with schizophrenia, with meta-analyses implicating trauma as one contributing etiological factor in the development of psychosis.^17^ ^,^^18^ ^,^^19^ ^,^^20^ Concerning first-episode psychosis, approximately one in two people experiences PTSD symptoms and one in three people experiences full PTSD following a first psychotic episode.^21^

Moreover, both schizophrenia and PTSD are associated with physical, mental, emotional, social, and cognitive dysfunction.^22^ Comorbid PTSD has been associated with poor social outcome, functioning, and quality of life.^23^ ^,^^24^ ^,^^25^ PTSD is also associated with a greater number of chronic physical health problems, including asthma, diabetes, hypertension, and cancer.^11^

Concerning correlates of comorbid PTSD in people with schizophrenia, no significant differences have been reported regarding sociodemographic variables, like age, ethnicity, nationality, education, employment, marital status, income, socioeconomic status, and living situation.^26^ ^,^^27^ ^,^^28^ Additionally, no differences have been reported regarding illness-related parameters, like age of schizophrenia onset,^27^ ^,^^29^ ^,^^30^ ^,^^31^ age of first hospitalization,^32^ ^,^^33^ number of hospitalizations^28^ ^,^^29^ ^,^^31^ ^,^^32^ substance and alcohol use disorders,^11^ ^,^^23^ and type of substance used.^28^ However, while there was also no significant difference between patients diagnosed with schizophrenia with versus without comorbid PTSD regarding antipsychotic prescriptions,^30^ ^,^^31^ ^,^^32^ a higher proportion of prescriptions for antidepressants has been reported in the comorbid PTSD group.^34^

With regard to clinical symptoms, results have been contradictory. While some studies report PTSD as being associated with more severe positive symptoms and less severe negative symptoms of schizophrenia, studies exploring both symptom domains within the same sample found no differences.^27^ ^,^^28^ ^,^^29^ ^,^^30^ ^,^^33^ ^,^^35^

Comorbid PTSD in people with schizophrenia is, however, linked to worse overall depressive symptom prevalence and severity. Significant correlations have been found between PTSD and a higher prevalence of comorbid depression as well as more severe forms of depression.^26^ ^,^^29^ ^,^^36^ ^,^^37^ Notably, suicidality was significantly associated with co-morbid PTSD, while suicidal behavior was directly correlated with the number of traumatic events.^28^

Furthermore, both PTSD and schizophrenia have been associated with persistent, low-degree inflammation,^37^ ^,^^38^ ^,^^39^ potentially contributing to poor psychiatric and medical outcomes. Studies have shown increased levels of inflammatory markers in schizophrenia.^40^ ^,^^41^ Concerning PTSD, although studies have sometimes yielded mixed results,^42^ recent trials have found elevated levels of several inflammatory markers in patients with PTSD.^38^ ^,^^43^ ^,^^44^

Easily accessible inflammation biomarkers, derived from full blood count variables, include the neutrophil-to-lymphocyte ratio (NLR), the platelet-to-lymphocyte ratio (PLR), and the systemic immune-inflammation index (SII: [platelets × neutrophils]/lymphocytes).^45^ SII is employed mainly in cancer, rheumatism, and immunity^28^ ^,^^46^ and has in recent years gained attention as an inflammatory marker for schizophrenia.^47^

There is clear evidence of under-reporting and insufficient screening for PTSD in people living with schizophrenia.^10^ ^,^^48^ ^,^^49^

The findings concerning the impact of comorbid PTSD on functionality and the relationship between PTSD and suicidality as well as cardiometabolic illness, underscore the critical importance of assessing trauma history as well as PTSD in the routine evaluation of patients with schizophrenia.

It is further necessary to update the available data since, to the best of our knowledge, there are no recent studies that have investigated the frequency and correlates of PTSD in people with schizophrenia. Importantly, this study was conducted in a low- and middle-income country setting, where exposure to environmental stressors and violence may be higher compared to high-income countries. This context may influence both trauma exposure and the prevalence of PTSD among individuals with schizophrenia, and should be considered when interpreting findings. Therefore, we carried out this study to fill this gap in the literature and generate data that can guide further clinical and research decisions.

## Methods

Cross-sectional, observational study to report the prevalence, severity and clinical as well as inflammatory correlates of PTSD among patients with schizophrenia. The study conducted in the outpatient service of a large public tertiary psychiatric hospital (Hospital José T. Borda, Buenos Aires, Argentina), which provides care primarily for patients with severe mental illness. Although all participants were recruited from outpatient settings, the clinical severity varied, including both relatively stable individuals and those with higher levels of symptomatology.

### Objectives

#### Primary objective

To assess the prevalence and severity of PTSD in adult outpatients with schizophrenia.

#### Secondary objectives

To explore the relationship in adults with schizophrenia with comorbid DSM-5 diagnosis PTSD (Mini-International Neuropsychiatric Interview (MINI) and: (i) sociodemographic factors (age, sex, marital status, employment status); (ii) age of onset of the schizophrenia disease; (iii) psychiatric comorbidities, including, depression, alcohol, and substance use disorders (MINI); (iv) depression severity (Calgary Depression scale for Schizophrenia (CDSS); (v) use of substances and alcohol; (vi) number and type of medical comorbidities; (vii) number of hospitalizations; (viii) severity of schizophrenia psychopathology (Positive and Negative Syndrome Scale [PANSS] total and subscores); (ix) suicidality (current suicidal thinking, number of lifetime suicide attempts); (x) functioning (Global Assessment of functioning scale (GAF); (xi) treatment-resistant schizophrenia (using the TRRIP criteria)^50^; xii) number and type of psychotropic medications (antidepressants, anxiolytics/hypnotics, mood stabilizers, antipsychotics, antipsychotic polypharmacy [cross-sectional and retrospective duration >/=60 days]); and (xiii) levels of inflammatory markers (NLR, PLR, SII).

### Inclusion and exclusion criteria

#### Inclusion criteria

Adult outpatients >/= 18 years old with a schizophrenia or schizophreniform disorder diagnosis (DSM-5) according to medical records and verified with the MINI.

#### *Exclusion* criteria

(i) Unconfirmed schizophrenia or schizophreniform disorder; (ii) any other axis I diagnosis (DSM-5) presenting as the principal diagnosis; (iii) unable to understand Spanish; (iv) unable to perform study related procedures, such as taking part in research interviews or filling our self-reports; and (v) unable to provide written informed consent.

### Assessments

The following data were registered as follows: age, sex, marital status, employment, age of onset of the disease, number of hospitalizations, current suicidal thinking, number of suicide attempts, current psychiatric medications, use of substances, and use of alcohol. Patients were administered the MINI^51^ to confirm the diagnosis of schizophrenia or schizophreniform disorder and the presence of PTSD as well as other psychiatric comorbidities, including substance use and alcohol use disorders. When PTSD was present, the Post-traumatic Stress Disorder Symptom Severity Scale-Revised (EGS-R) scale was used to assess severity.^52^ All patients were administered the PANSS,^53^ CDSS,^54^ and GAF.^55^

Hemograms with date of blood draws from the last 3 months were retrieved from medical records to obtain NLR, PLR, and SII. If no full blood count was available in the charts from </= 3 months ago, blood was drawn to obtain these data.

### Statistical analysis

#### Descriptive statistics

Categorical data were expressed as frequency and percentage, continuous variables were expressed as mean and SD when normally distributed, and as median with interquartile range when non-normally distributed.

#### Bivariate analysis

Chi-square test or Fisher’s exact test was used to examine the relationship between categorical variables and PTSD comorbidity. P-values and effect sizes were reported. For continuous variables with normal distribution an independent t-test was used, for non-normally distributed variables or Mann–Whitney *U* test was used.

#### Correlation analysis

To examine the relationship between PTSD severity and PANSS/CDSS/GAF scores and levels of inflammatory markers, Pearson’s correlation analyses for normally distributed variables or Spearman’s rank correlation for non-normally distributed variables was used.

#### Multivariable analysis

Multivariable backward elimination logistic regression analysis was used to assess the relationship between the assessed categorical and continuous variables and the likelihood of having PTSD. Variables entered into the initial model were those significant at p < 0.05 selected from an omnibus test comparing all noncollinear variables in patients with versus without comorbid schizophrenia.

### Sample size and power calculation

For the primary, descriptive objective, the estimation of the prevalence and severity of PTSD in adult outpatient with schizophrenia, we set an arbitrary minimum number of 200 patients, as 200 subjects will provide sufficiently precise estimates of the prevalence of PTSD. To assess the statistical power for the analyses of correlates of PTSD in the proposed study, we considered two primary types of analyses: (i) comparisons of categorical variables and (ii) comparisons of continuous variables to distinguish between patients with schizophrenia with and without comorbid PTSD. Below, we describe the methodology and assumptions used to estimate the power for each type of analysis.

#### Study *design* and assumptions

- Total sample size: 200 subjects with schizophrenia.
- Subgroup distribution: 25% of subjects (n = 50) are expected to have comorbid PTSD, and 75% (n = 150) will not.
- Significance level (α): 0.05 (two-sided analyses).
- Effect sizes: Assumed based on clinically relevant differences between groups, informed by prior studies or pilot data.

#### *Power* for categorical variables

For categorical variable analyses (eg, the presence/absence of a specific symptom), we used a chi-squared test to estimate power under varying assumptions about group differences.

- Expected proportions: Assuming a clinically relevant difference of 20% between groups in the prevalence of a specific categorical variable (eg, 50% in the PTSD group vs. 30% in the non-PTSD group).
- Effect size (Cohen’s w): Calculated as $w=\sqrt{\sum {\frac{\left(O-E\right)}{E}}^{2}}$ where O and E are the observed and expected proportions, respectively.
- Power estimate: With n = 50 in the PTSD group and n = 150 in the non-PTSD group, we estimate the power to detect this difference to be approximately 80% (calculated using G*Power or similar statistical software).

#### Power for *continuous* variables

For continuous variable analyses (eg, symptom severity scores), we used a two-sample t-test to compare means between groups.

- Effect size (Cohen’s d): Assumed to range from small (d = 0.3) to medium (d = 0.5) based on prior studies.
- Power estimates:

◦For a small effect size (d = 0.3), power is approximately 55%.

◦For a medium effect size (d = 0.5), power increases to approximately 87%.

These calculations assume equal variances across groups and a two-sided test at α = 0.05).

Unequal variances or deviations from normality may impact power and would be addressed through sensitivity analyses or nonparametric methods. Thus, the proposed sample size of 200 subjects provides sufficient power for detecting medium to large effect sizes in both categorical and continuous variable analyses. For small effect sizes, the study may be underpowered, and findings would need to be interpreted cautiously. Additional exploratory analyses may help identify trends warranting further investigation in larger samples.

### Ethical considerations

The study was conducted in compliance with the protocol, the Declaration of Helsinki, ICH GCP, and other applicable regulatory requirements. The ethics committee of the Hospital Tobar García approved this study (study number: 16377). Written informed consent was obtained from each subject prior to any screening or study procedures.

## Results

The analysis includes data from 205 patients diagnosed with schizophrenia who were enrolled in the ongoing study. All participants provided informed consent prior to inclusion. Demographic information, medical, and psychiatric history were collected through medical records and structured clinical interviews.

Participants then underwent standardized clinical assessments.

Among the sample, 190 (92.7%) were male and 15 (7.3%) were female, with a mean age of 42.4 ± 13.4 (range = 18–79) years. Regarding marital status, 95.1% were single, 2.9% were married, 1.5% were divorced, and 0.5% were widowed. Employment status indicated that 82.9% were unemployed, 14.2% worked informal jobs, and 2.5% held formal employment, while 0.5% were currently studying.

The mean age of illness onset was 20.8 ± 6.9 (range = 9–44) years. Patients had a mean of 2.2 ± 2.9 hospitalizations (range = 0–16). A history of suicide attempts was reported in 27.3% of patients, with a mean of 1.7 ± 1.3 attempts (range = 0–5). No current suicidal ideation was registered. Regarding substance use, 8.8% reported current alcohol consumption and 2.9% reported active use of other substances. A total of 60.0% had no medical comorbidities, while 40.0% had at least one medical condition.

The mean number of medical comorbidities was 0.74 ± 1.09 (range = 0–5), the most common being: prediabetes (42.7%), dyslipidemia (37.8%), hypertension (31.7%), and diabetes (17%). Treatment resistance, defined using TRRIP criteria, was present in 36.6% of the sample. A summary of the patient sample data is presented in Table 1.

**Table 1.** Summary of patient, illness, and treatment characteristics Table 1. long description.

Characteristic	Total (n = 205)	Schizophrenia without PTSD comorbidity (n = 163)	Schizophrenia with PTSD comorbidity (n = 42)	Test	p-value
Age (years), mean ± SD	42.4 ± 13.4	42.1 ± 13.7	43.5 ± 12.1	t(203) = 0.60	.600
Male sex (%)	92.7%	92.6%	92.9%	χ^2^(1) = 0.00	.961
Marital status (%)				χ^2^(3) = 4.27	.234
Single	95.1%	95.7%	92.9%		
Married	2.9%	1.8%	7.1%		
Divorced	1.5%	1.5%	0.0%		
Widow/er	0.5%	0.6%	0.0%		
Employment (%)				χ^2^(3) = 1.60	.660
Unemployed	82.9%	82.2%	85.7%		
Informally employed	14.2%	14.1%	14.3%		
Formally employed	2.5%	3.0%	0.0%		
Student	0.5%	0.6%	0.0%		
Age at illness onset (years), mean ± SD	20.8 ± 6.9	20.8 ± 6.9	21.2 ± 6	t(203) = 0.35	.463
Number of hospitalizations, mean ± SD	2.2 ±	2.1 ± 2.9	2.2 ± 3.3	t(203) = 0.35	.463
History of suicide attempts (% yes)	27.3%	23.0%	40.5%	χ^2^(2) = 5.98	.050
Mean number of suicide attempts, mean ± SD	1.7 ± 1.3	1.5 ± 1.1	2.4 ± 1.2	t(203) = 1.94	.066
Medical comorbidities (% yes)	45.2%	42.8%	51.1%	χ^2^(1) = 6.47	.014
Number of medical comorbidities, mean ± SD	0.7 ± 1.1	0.6 ± .1.0	1.1 ± 1.3	t(203) = 3.56	.010
Substance use (% yes)	2.9%	2.5%	4.8%	χ^2^(1) = 0.63	.429
Alcohol use (% yes)	8.8%	6.8%	16.7%	χ^2^(1) = 4.10	.062
Depression (% yes)	33.7%	23.3%	73.8%	χ^2^(1) = 38.14	<.001
CDSS score, mean ± SD	4.4 ± 4.8	3.5 ± 4.1	8.9 ± 4.9	t(203) = 7.68	<.001
PANSS total score, mean ± SD	73.5 ± 17.9	71.6 ± 17.1	80.7 ± 19.5	t(203) = 7.68	.003
PANSS positive subscale score, mean ± SD	15.6 ± 6.1	14.9 ± 5.7	17.9 ± 6.7	t(203) = 2.74	.005
PANSS negative subscale score, mean ± SD	24.3 ± 6.4	23.4 ± 6.5	25.5 ± 5.9	t(203) = 1.30	.168
PANSS general psychopathology subscale score, mean ± SD	34 ± 8.9	32.5 ± 8.4	40.2 ± 8.8	t(203) = 5.23	<.001
GAF score, mean ± SD	47.6 ± 12.9	49.6 ± 12.3	39.8 ± 12.3	t(203) = 3.86	<.001
Treatment resistance (% yes)	36.6%	37.4%	33.3%	χ^2^(1) = 0.24	.624
Concomitant medication (%)	60.4%	53.4%	78.6%	χ^2^(2) = 9.27	.010
Number of concomitant medications, mean ± SD	1.2 ± 1.1	1.0 ± 1.0	1.7 ± 1.1	t(203) = 3.56	<.001
Antipsychotic polypharmacy (%)	9.3%	9.2%	9.5%	χ^2^(1) = 0.00	.949
NLR, mean ± SD	2.1 ± 1.0	2.0 ± 0.8	2.4 ± 1	t(203) = 1.57	.133
PLR, mean ± SD	120.2 ± 53.7	120.1 ± 54.0	120.5 ± 54.3	t(203) = 1.51	.444
SII, mean ± SD	713.2 ± 561.9	672.8 ± 510.4	814.1 ± 623.4	t(203) = 1.35	.180

### Current antipsychotic treatment

The distribution of antipsychotic treatment was as follows:

Risperidone: 42.9%; clozapine: 32.2%; haloperidol decanoate: 4.4%; aripiprazole: 4.4%; haloperidol: 3.4%; risperidone + haloperidol: 2.9%; olanzapine: 2.4%; risperidone + clozapine: 1.5%; aripiprazole + risperidone: 0.9%; haloperidol + clozapine: 0.9%; risperidone + haloperidol decanoate: 0.5%; aripiprazole + clozapine: 0.5%; quetiapine: 0.5%; risperidone + quetiapine: 0.5%; and risperidone + olanzapine: 0.5%. Antipsychotic polypharmacy was present in 9.3% of patients.

### Concomitant psychotropic medication

Concomitant psychotropic medication was present in 60.4% of patients. Mean number of concomitant medications was 1.16 ± 1.06 (range = 0–4). Distribution of concomitant medications was as follows: 52.2% benzodiazepines (lorazepam 42.9%; clonazepam 8.3%; diazepam 0.5%; alprazolam 0.5%), 35.1% mood stabilizers (sodium divalproate 25.8%; lamotrigine 4.9%; carbamazepine 3.4%; lithium 0.5%; oxcarbamazepine 0.5%), 17.6% biperiden, 13.7% antidepressants (paroxetine 4.9%; sertraline 3.8, fluoxetine 2%; citalopram 1.5%, venlafaxine 1.5%).

### Prevalence of PTSD and clinical measures

PTSD was diagnosed in 20.5% of patients using the MINI. For these patients, the EGS-R scale yielded a mean score of 23.7 ± 10.9 (range = 10–52). Psychopathology assessments yielded the following mean scores: PANSS total: 73.5 ± 17.9 (range = 6–134), PANSS Positive subscale score: 15.6 ± 6 (range = 7–33), PANSS Negative subscale: 24.3 ± 6.4 (range = 8–42), PANSS General Psychopathology subscale: 34 ± 8.9 (range = 12–68). GAF mean score for the sample was 47.6 ± 12.9 (range = 20–85). Concerning the CDSS, the mean score was 4.4 ± 4.8 (range = 0–18), with 33.7% of patients meeting criteria for depression (ie, ≥ 6) (Addington et al, 1992).

### Inflammatory markers

Data for the NLR, PLR, and SII were available for 42.2% of patients. Across the total sample, the mean NLR was 2.12 ± 0.96, mean PLR was 120.15 ± 53.73, and mean SII was 713.2 ± 561.9.

### Childhood-onset schizophrenia

A total of 15 participants (7.3%) had an age of onset ≤13 years, consistent with childhood-onset schizophrenia. In this subgroup, the prevalence of PTSD was 6.7%. Due to the small sample size, no formal statistical comparisons were conducted, and these findings should be interpreted as exploratory.

### Bivariate analyses

**Figure 1.**
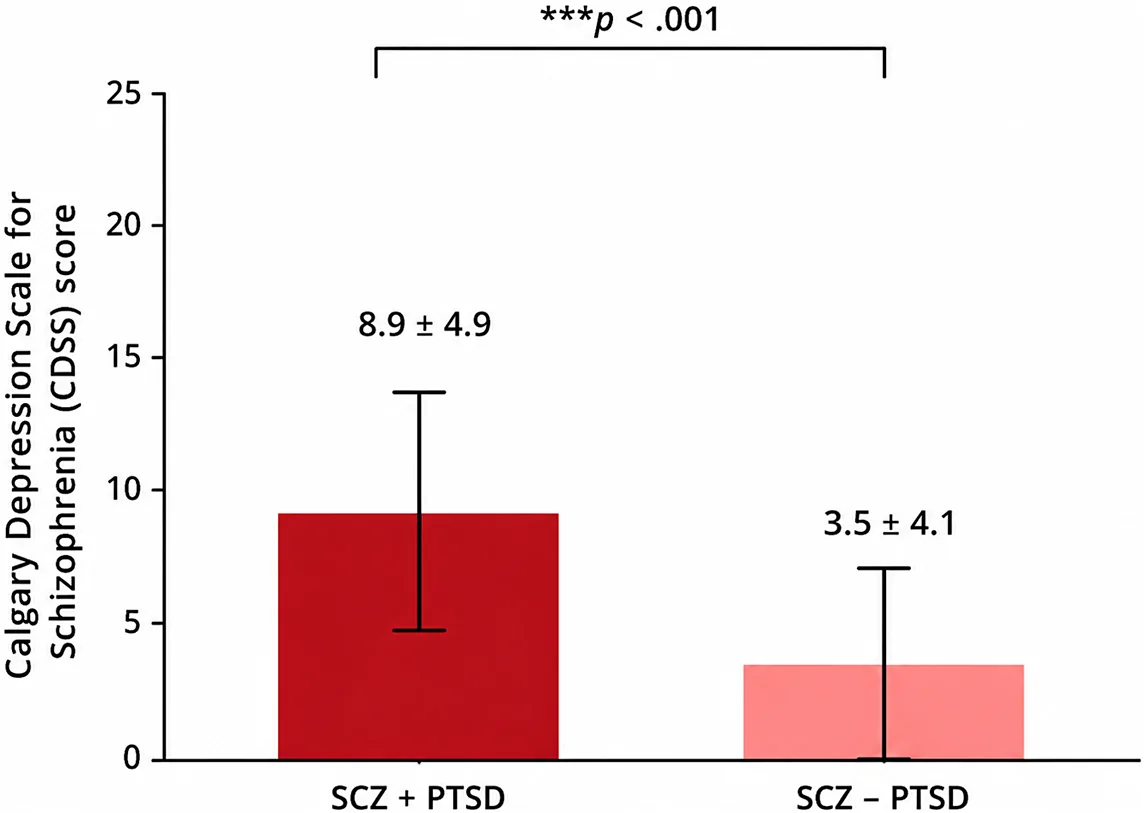
Depression severity (CDSS score shown as mean and standard deviation) by PTSD status. CDSS: Calgary depression scale for schizophrenia; PTSD: post-traumatic stress-disorder; SCZ: schizophrenia.

The bivariate analyses showed that both presence of medical comorbidities (SCZ + PTSD:51.1% vs. SCZ-PTSD:42.8%, p = .014), and the mean number of comorbidities (1.1 ± 1.3 vs. 0.6 ± .1.0, p = .010) were associated with PTSD. Depression was more prevalent (73.8% vs. 23.3%, p < .001) and CDSS scores were higher in the PTSD group compared to the non-PTSD group (8.9 ± 4.9 vs. 3.5 ± 4.1, p < .001) (Figure 1).

**Figure 2.**
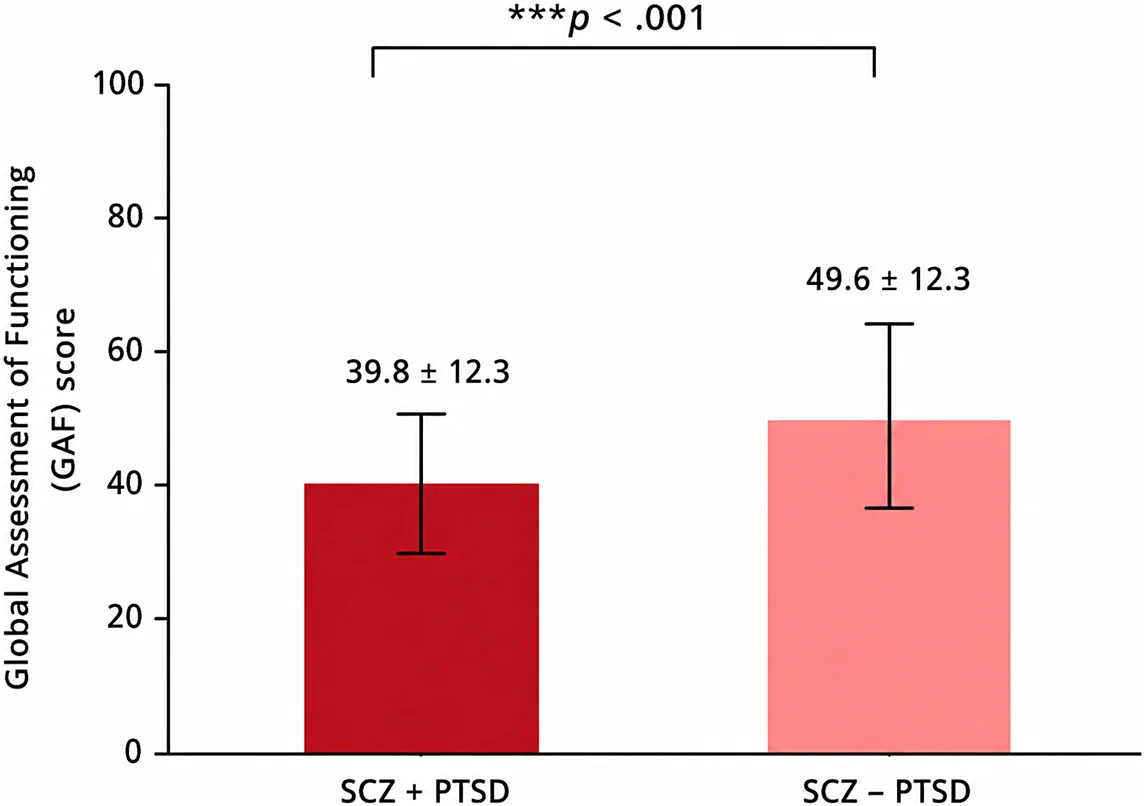
Functionality (GAF score shown as mean and standard deviation) by PTSD status. GAF: Global assessment of functioning; PTSD: post-traumatic stress-disorder; SCZ: schizophrenia.

Psychotic symptom severity was also greater in patients with PTSD, with significantly higher PANSS total scores (80.7 ± 19.5 vs. 71.6 ± 17.1, p = .003). This difference was evident in both the Positive subscale (17.9 ± 6.7 vs. 14.9 ± 5.7, p = .005) and the General Psychopathology subscale (40.2 ± 8.8 vs. 32.5 ± 8.4, p < .001). Patients with PTSD had significantly lower GAF scores (39.8 ± 12.3 vs. 49.6 ± 12.3, p < .001) (Figure 2).

In regard to treatment variables, patients with PTSD had more frequently concomitant medication (51.1% vs. 42.8%,p = .010) and a higher mean number of concomitant medications (1.7 ± 1.1) compared with those without PTSD (1.0 ± 1.0, p < .001).

In contrast, no significant differences were found between groups in demographic variables such as age, sex, marital status, occupation, or age at illness onset. Substance abuse, previous suicide attempts, and the number of hospitalizations were also not significantly significant.

Within clinical variables, differences in the PANSS Negative subscale were modest and did not reach statistical significance. Similarly, inflammatory markers did not differ significantly between groups: although NLR (2.42 ± 1 vs. 2.0 ± 0.8) and SII (814.1 ± 623.4 vs. 672.8 ± 510.4) were numerically higher in the PTSD group, these differences were not statistically significant, and PLR values were nearly identical between groups (120.53 ± 54.32 vs. 120.05 ± 53.98). Finally, polypharmacy and treatment resistance showed no association.

### Stepwise backward elimination logistic regression analysis

The initial logistic regression model included variables that were found to be statistically significant in the bivariate analyses. These variables were: medical comorbidities, presence of depression, CDSS rating, PANSS total score, PANSS positive subscale score, PANSS general psychopathology score, GAF score, and concomitant medication.

After model refinement, the final logistic regression model retained only two significant predictors for the presence of PTSD: Depression severity (measured using CDSS) and GAF score. (χ^2^(2) = 51.50, p < .001) The model explained a meaningful proportion of variance in PTSD status (Cox & Snell R^2^ = 0.22, Nagelkerke R^2^ = 0.35, McFadden R^2^ = 0.25) Higher Calgary Depression Scale scores were significantly associated with an increased likelihood of PTSD. Each one-point increase in Calgary score was associated with a 25% increase in the odds of PTSD (OR = 1.25, 95% CI [1.15, 1.36], p < .001).

Lower GAF scores were significantly associated with PTSD: for each one-point reduction in GAF score, the odds of PTSD increased by approximately 4% (OR = 0.96, 95% CI [0.93, 0.99], p = .007).

## Discussion

This analysis includes a clinically well-characterized sample of outpatients with schizophrenia from a tertiary psychiatric hospital, allowing the exploration of associations between PTSD and demographic, clinical, and biological variables. There was an overwhelming male predominance since the psychiatric institution where the study carried out attends predominantly male patients.

The majority of participants were unemployed (82.9%) and single (95.1%), consistent with prior findings in populations with schizophrenia, particularly in tertiary-care settings where individuals with greater illness severity and functional impairment are more likely to receive care.

The prevalence of PTSD in our sample was 20.5%, which is in line with previously reported rates ranging from 10 to 30% in patients with schizophrenia, depending on the population and methodology used.^7^ ^,^^10^ ^,^^11^ ^,^^13^ This prevalence exceeds that observed in the general population,^6^ ^,^^14^ suggesting that individuals with schizophrenia may be both more frequently exposed to trauma and more vulnerable to developing PTSD. Interestingly, exploratory analyses suggested a lower prevalence of PTSD among patients with childhood-onset schizophrenia. However, given the small number of cases, this finding should be interpreted with caution and warrants further investigation in larger samples.

Patients with PTSD were more likely to have one or more medical conditions, which correlates with existing findings^11^ and a higher mean number of medical comorbidities. Most frequent comorbidities included hyperglycemia, dyslipidemia, and hypertension.

In our sample, patients with PTSD presented significantly more severe symptoms as measured by the PANSS total score, particularly in the Positive and General Psychopathology subscales. Which may reflect increased exposure to trauma and/or vulnerability to PTSD in this population.^56^ The fact that the PANSS Negative subscale did not differ significantly suggests that trauma may be more closely related to affective and positive symptoms rather than negative symptoms per se. However, these findings should be interpreted in the context of the study setting and may not be generalizable to all individuals with schizophrenia.

In our sample, patients with PTSD presented significantly more severe symptoms as measured by the PANSS total score, particularly in the Positive and General Psychopathology subscales. These findings are in line with prior work indicating that trauma exposure may exacerbate psychotic symptoms or that psychosis may increase vulnerability to traumatic stress.^56^ The fact that the PANSS Negative subscale did not differ significantly suggests that trauma may be more closely related to affective and positive symptoms rather than negative symptoms per se.

Individuals diagnosed with comorbid PTSD showed significantly lower levels of global functioning, as measured by the GAF scale. The nearly 10-point difference in GAF scores suggests that PTSD may contribute meaningfully to additional disability beyond the primary psychiatric diagnosis. These results are consistent with previous research indicating that PTSD is associated with poorer psychosocial outcomes.^24^ ^,^^25^

The strong association between PTSD and depressive symptoms in our study is also consistent with a robust body of literature. Then, 73.8% of patients with PTSD met criteria for depression, and their mean Calgary score was almost three times higher than those without PTSD. This finding mirrors previous reports.^29^ ^,^^36^ ^,^^57^The PTSD group also showed a higher rate of suicide attempts, consistent with findings that trauma-exposed individuals with schizophrenia are at elevated risk for suicidal behavior.^28^

On the other hand, demographic factors such as age, sex, and marital, employment status, use of antidepressants and substance use were not significantly associated with PTSD, although group sizes in some cases were too small which limited statistical power.

Also, no significant associations were found between PTSD and treatment resistance or antipsychotic polypharmacy. However, these results should be interpreted with caution, due to the cross-sectional nature of the study.

Exploration of inflammation through NLR, PLR, and SII did not reveal statistically significant differences between groups. This was unexpected, given that elevated inflammatory markers have been observed both in PTSD^38^ ^,^^43^ ^,^^44^ and schizophrenia.^40^ ^,^^41^ However, it has to be taken into account that these indicators are subject to the effect of multiple factors including systemic inflammation, stress, and metabolic dysfunction. Also, the model sample size and the absence of stratification by factors, such as obesity, age or medication limits the interpretability of such results.

The logistic regression analyses identified key factors associated with comorbid PTSD in patients with schizophrenia. Importantly, our regression analyses identified two robust predictors of PTSD: depression severity, measured with the CDSS, and functional impairment, measured with the GAF. Specifically, patients with lower GAF scores were significantly more likely to present with PTSD. This indicates that beyond depressive symptomatology, diminished psychosocial functioning is a critical clinical marker of comorbid PTSD in schizophrenia. Together, these findings underscore that the burden of PTSD in schizophrenia is characterized not only by more severe depression but also by greater disruption in daily life and functioning, reinforcing the need for systematic screening of trauma and PTSD in routine clinical practice.

### Limitations

An important consideration is the composition of the sample. While the overall sample size of 205 patients is adequate, the relatively small number of participants in subgroups limits the statistical power for detecting more subtle associations.

Additionally, participants were mainly male, which reduces the generalizability of findings to women with schizophrenia who may present distinct clinical trajectories, response to treatment and comorbidities. This raises the important questions about gender-specific factors in schizophrenia and PTSD. Also, the small number of patients with childhood-onset schizophrenia limited the ability to perform robust subgroup analyses.

Furthermore, the study was conducted in a public psychiatric hospital setting, where patients are typically from low-resource backgrounds. This may also limit generalizability of findings to more socioeconomically diverse populations. Finally, the cross-sectional nature of the study restricts causal inference.

## Conclusions

The findings emphasize the high prevalence and clinical significance of PTSD in patients with schizophrenia. PTSD was present in one in five patients with schizophrenia and was linked to greater clinical burden, including more severe depressive and psychotic symptoms, higher suicidality, and poorer functioning. Depression severity and lower GAF scores were the strongest predictors of PTSD, underscoring the need for routine screening and targeted interventions to improve outcomes in this population.

## Long descriptions

### Table 1. Long description

The table consists of 6 columns: Characteristic, Total (n = 205), Schizophrenia without P T S D comorbidity (n = 163), Schizophrenia with P T S D comorbidity (n = 42), Test, and p-value.

Key findings include:

* Age: Mean age is approximately 42 to 43 years across all groups (p = .600).

* Sex: Predominantly male (over 92% in all groups).

* History of suicide attempts: Significantly higher in the P T S D group (40.5%) compared to the non-P T S D group (23.0%) with a p-value of .050.

* Depression: Highly prevalent in the P T S D group (73.8%) versus the non-P T S D group (23.3%) with p < .001.

* C D S S score: Significantly higher in the P T S D group (8.9 plus or minus 4.9) than the non-P T S D group (3.5 plus or minus 4.1) with p < .001.

* P A N S S total score: Higher in the P T S D group (80.7 plus or minus 19.5) compared to the non-P T S D group (71.6 plus or minus 17.1) with p = .003.

* G A F score: Lower in the P T S D group (39.8 plus or minus 12.3) compared to the non-P T S D group (49.6 plus or minus 12.3) with p < .001.

* Medication: The P T S D group used more concomitant medications (1.7 plus or minus 1.1) than the non-P T S D group (1.0 plus or minus 1.0) with p < .001.

* Inflammatory markers: N L R, P L R, and S I I showed no statistically significant differences between groups (p-values of .133, .444, and .180 respectively).

Navigate back to Table 1..
